# Snake trajectories in ultraclean graphene p–n junctions

**DOI:** 10.1038/ncomms7470

**Published:** 2015-03-03

**Authors:** Peter Rickhaus, Péter Makk, Ming-Hao Liu, Endre Tóvári, Markus Weiss, Romain Maurand, Klaus Richter, Christian Schönenberger

**Affiliations:** 1 Department of Physics, University of Basel, Klingelbergstrasse 82, CH-4056 Basel, Switzerland; 2 Institut für Theoretische Physik,Universität Regensburg, D-93040 Regensburg, Germany; 3 Department of Physics, Budapest University of Technology and Economics and Condensed Matter Research Group of the Hungarian Academy of Sciences, Budafoki ut 8, 1111 Budapest, Hungary; 4 University Grenoble Alpes, CEA-INAC-SPSMS, F-38000 Grenoble, France

## Abstract

Snake states are trajectories of charge carriers curving back and forth along an interface. There are two types of snake states, formed by either inverting the magnetic field direction or the charge carrier type at an interface. The former has been demonstrated in GaAs–AlGaAs heterostructures, whereas the latter has become conceivable only with the advance of ballistic graphene where a gap-less p–n interface governed by Klein tunnelling can be formed. Such snake states were hidden in previous experiments due to limited sample quality. Here we report on magneto-conductance oscillations due to snake states in a ballistic suspended graphene p–n junction, which occur already at a very small magnetic field of 20 mT. The visibility of 30% is enabled by Klein collimation. Our finding is firmly supported by quantum transport simulations. We demonstrate the high tunability of the device and operate it in different magnetic field regimes.

## Abstract (web summary)

Snake states describe electron trajectories that curve along an interface where the charge is inverted. Here, the authors investigate electronic transport in a ballistic graphene p–n junction and observe striking conductance oscillations that are a signature of these unusual states.

## Graphical abstract

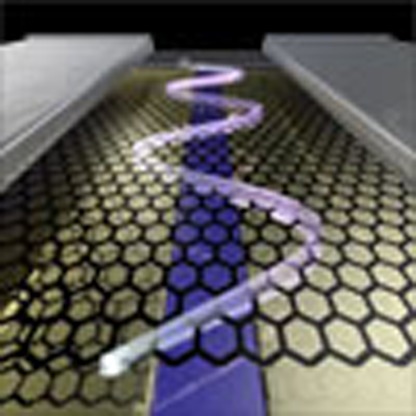

A magnetic field fundamentally modifies the transport properties of an electronic conductor by acting on its charge carriers via the Lorenz force. The most prominent magnetotransport effect is the quantum Hall effect in a two-dimensional electron gas. A strong perpendicular magnetic field forces the charge carriers into one-dimensional conduction channels in which they flow along the edges of a sample. At moderate magnetic fields, however, electron trajectories can be understood in a quasiclassical picture where the Lorenz force bends charge carriers into cyclotron orbits. In the bulk this leads to localization, whereas at the boundary charge carriers can propagate via so-called skipping orbits. Magnetic focusing experiments^1^,^2^ represent a direct proof of the skipping orbit picture. In such experiments, an increase of conductance is observed if the distance between two contacts is an integer multiple of the diameter of a cyclotron orbit. One condition for the observation of such trajectories is ballistic transport over the relevant device dimensions. This has limited the observation of skipping orbits to the cleanest available semiconductor samples.

Since 2004, graphene as a new two-dimensional conductor has moved into the focus of condensed-matter research and its behaviour in magnetic field has been intensely investigated. The quality of graphene devices has improved over the recent years and ballistic transport over distances of several microns have been demonstrated recently^3^,^4^. Since graphene is a gap-less semiconductor, it offers the possibility of creating internal interfaces with opposite charge carrier polarity. These so-called p–n interfaces^5^,^6^,^7^ are formed by local electrostatic gating.

If electrons that propagate via skipping orbits encounter such a p–n interface, they will turn into snake states. These states consist of alternating half circles with opposite chirality and they transport current along the interface. Similar snake states have first been realized in GaAs/AlGaAs two-dimensional electron gases by defining regions of alternating magnetic field direction^8^. These states share the condition of commensurability (similar to the above-described magnetic focusing experiments) with p–n snake states but they do not propagate along a single and tunable interface. Snake states in graphene p–n junctions were claimed to have been observed in disordered substrate-supported samples^9^ but the experiment lacked direct evidence for snaking trajectories.

In this article, we investigate ballistic transport across a graphene p–n junction in different magnetic field regimes and identify magneto-conductance oscillations as a direct signature of snake states. These findings are supported by detailed tight-binding simulations that allow us to visualize the alternating cyclotron orbits of the snake states.

## Results

### Evolution of electron trajectories in graphene p–n junction

**Figure 1:**
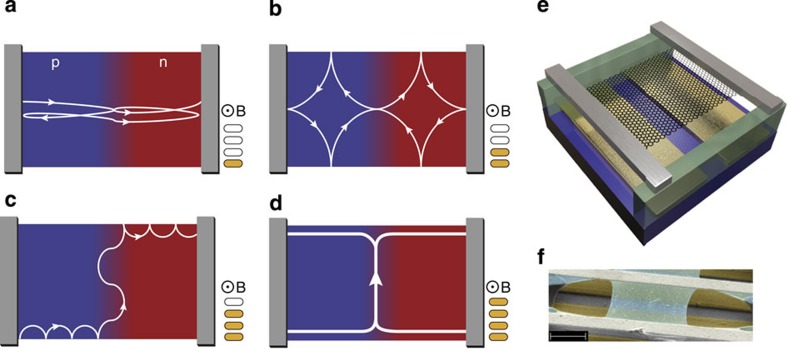
Evolution of electron states in increasing magnetic field and design of a graphene p–n junction. (**a**) A two-terminal graphene device consisting of a hole (blue) and an electron (red) cavity is sketched. By applying a weak field, the electron trajectories in the p- and n-cavities bend, leading to dispersing Fabry–Pérot resonances. (**b**) The field is increased until the cyclotron orbit becomes comparable to the cavity size, where resonant scar states can occur. (**c**) The field is further increased and transport is still described by quasiclassical cyclotron orbits. Snake states are formed along the p–n interface. (**d**) Finally, quantum Hall edge states propagate in opposite directions in the p- and n-region at higher fields. (**e**) Three-dimensional design of the measured device. The SiO_2_ substrate is coloured in blue and the bottom gates in gold. The contacts, supported by the lift-off resist (green) are coloured in grey. (**f**) Scanning electron microscope image of a device similar to the measured sample. The graphene is coloured in blue and the bottom gates in gold. Scale bar, 1 μm.

In Fig. 1a–d, we illustrate schematically how trajectories under increasing perpendicular magnetic field evolve in such a device. Figure 1a describes the low-field situation where transport is still dominated by Fabry–Pérot oscillations with slightly bent trajectories^10^. As the field is increased, resonant scar states (Fig. 1b) may occur, as observed in semiconductor quantum dots^11^. At higher fields (Fig. 1c), snake states at the p–n interface govern the electronic properties. Finally (Fig. 1d) the system enters the quantum Hall regime^12^,^13^ where transport is dominated by edge states and Landau level mixing at the p–n interface can occur^14^,^15^. Even though this article is focusing on snake states, we will discuss the phenomenology of the mentioned magnetic field regimes. By doing so, we present an integral picture of graphene p–n physics in magnetic field.

### Device architecture

Figure 1e shows the design of the measured device and Fig. 1f a scanning electron microscope picture of a similar sample. In a suspended 2 × 2 μm graphene sheet, a p–n junction is formed by applying different voltages on the left (*V*_left_) and right (*V*_right_) bottom gates, resulting in different charge carrier concentrations *n*_left_ and *n*_right_. The fabrication follows partly ref. 16, which we combined with a wet transfer process allowing us to align the graphene with the bottom gates. For details, see ref. 17 and Methods.

### Measurements in the Fabry–Pérot and quantum Hall regime

**Figure 2:**
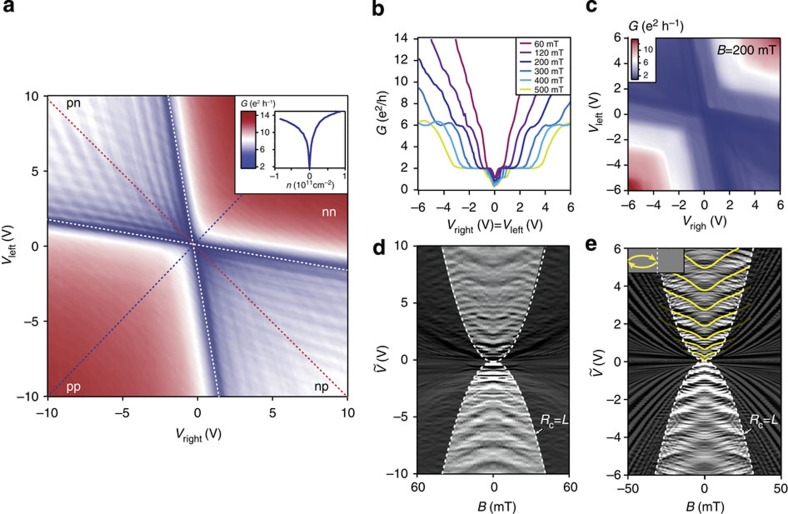
Device characterization in the Fabry–Pérot and in the quantum Hall regime. (**a**) Two-terminal conductance as a function of left and right gate voltage shows regular Fabry–Pérot oscillations at zero magnetic field. The inset reveals the narrow Dirac dip along the pp–nn diagonal (blue dashed line) from which a mobility of *μ*≈470 × 10^3^ cm^2^ V^−1^ s^−1^ is deduced. (**b**) Cuts along the same diagonal at different magnetic field strengths exhibit the expected quantum Hall plateaus at 2, 6, 10 *e*^2^/*h*. The *G*=2*e*^2^/*h* plateau is already visible at 60 mT. (**c**) The colour plot as a function of *V*_left_ and *V*_right_ at 200 mT shows quantum Hall plateaus in the unipolar region. (**d**) The numerical derivative 
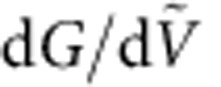
 (in arbitrary units) is recorded as a function of gate voltage 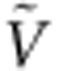 and *B* displaying the dispersion of the Fabry–Pérot oscillations. 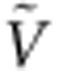 is the magnitude of gate voltage in the situation of antisymmetric charge densities *n*_left_=−*n*_right_ (red dashed line in **a**, that is, the np–pn diagonal). The white dashed curve indicates the line along which the cyclotron radius *R*_c_ is equal to the cavity length *L*=0.8 μm, the region *R*_c_<*L* is darkened and will be discussed in the main text. (**e**) The measured pattern is reproduced by a tight-binding quantum transport calculation based on the designed geometry of the measured device. The small inset shows a resonant electron trajectory at low magnetic field. Constructive interference occurs if the phase along this trajectory is an integer of 2*π*, leading to the numerical solution of the yellow lines (see Methods).

In the following, we characterize the measured device in the zero, low- and high-field regimes. Figure 2a shows a two-dimensional colour map of the electrical conductance *G*(*V*_right_,*V*_left_). As soon as a p–n interface is formed, *G* is lowered drastically. Regular Fabry–Pérot resonances in the left/right cavity are visible as oscillations perpendicular to the zero density line in the left/right cavity (horizontal/vertical white dashed line in Fig. 2a), indicating ballistic transport^3^,^4^. The inset in Fig. 2a is a slice along the pp–nn diagonal (blue dashed). We estimate the mobility to be 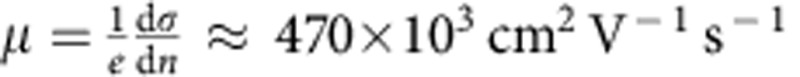 at carrier density *n*=1.1 × 10^9^ cm^−2^, which we calculated by a simple parallel plate capacitor model. The mobility is mainly limited by scattering at the contacts^3^,^18^.

In Fig. 2b, we show cuts along the pp–nn diagonal at different magnetic fields *B* and obtain quantum Hall plateaus at *G*=*G*_0_ × *ν*, where *ν*=2,6,10,... is the filling factor^12^,^13^ and *G*_0_=*e*^2^/*h* is the conductance quantum. Even at fields as low as 60 mT, the *ν*=2 plateau is visible. The colourscale map in Fig. 2c taken at 200 mT shows that plateaus develop in the unipolar region with conductance values given by the lowest number of edge modes in the left or right cavity, that is, *G*=*G*_0_ × min(*ν*_right_,*ν*_left_)=2, 6, 10 *e*^2^/*h*. This observation compares well to experiments of ref. 14, even though we apply only 0.2 T instead of 4 T. In the bipolar region, the conductance stays well below 2*e*^2^/*h* due to the smoothness of the p–n interface.

In a further step, we study the dispersion of the Fabry–Pérot interference pattern in low magnetic field^10^. Figure 2d shows the numerical derivative 
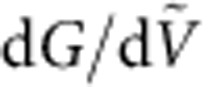
 as a function of *B* and 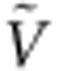, where 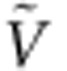 represents the magnitude of gate voltage in the situation of antisymmetric charge density (red dashed line in Fig. 2a, that is, the np–pn diagonal). In this configuration, the device consists of two Fabry–Pérot cavities of equal length *L*≈0.8 μm. The darkened region where the cyclotron radius *R*_*c*_<*L* will be discussed later. In Fig. 2e, a tight-binding transport calculation is shown, which reproduces the measured interference pattern very well (for details see ref. 19 and Methods). We highlight the quality of the measured graphene and the ability of the simulation to capture the complex oscillation pattern of this micron-sized system in the magnetic field. The dispersion of the Fabry–Pérot oscillations can be described by bent electron trajectories such as the one sketched in the inset of Fig. 2e (refs 3, 10). The condition for constructive interference is met if the accumulated phase along such a trajectory is a multiple of 2*π* (see ‘Methods’). The yellow lines in Fig. 2e are numerical solutions based on such a condition.

### Measurement of snake states

**Figure 3:**
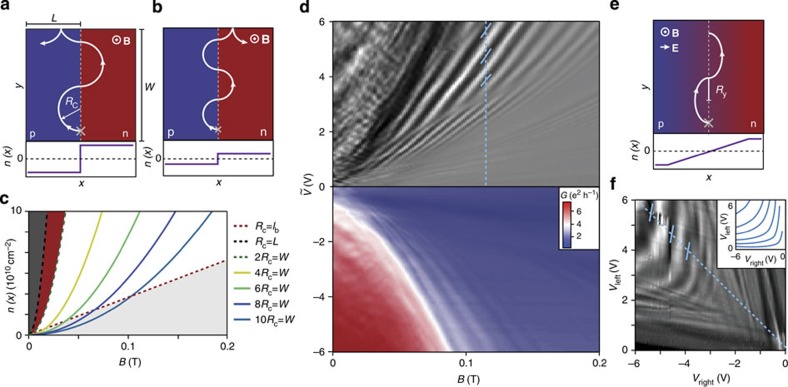
Parabola-like conductance oscillations as a signature of snake states. (**a**) Charge carrier trajectory (white) along a sharp p–n junction in perpendicular magnetic field starting at the grey cross, where *R*_c_ is the cyclotron radius and *n*(*x*) is the electron density. (**b**) At lower p- and n-density *R*_c_ is reduced. In contrast to **a**, the trajectory results in current flow towards the right contact. (**c**) Curves of constant *R*_c_ as a function of *n* and *B*. The continuous lines are given by the condition that the cyclotron diameter 2*R*_c_ is commensurate with the sample width *W*. Snake states occur between the green dashed 2*R*_c_=*W* and the red dashed *R*_c_=*l*_*B*_ line. The black dashed line indicates up to which field transport is dominated by bent Fabry–Pérot patterns (dark grey area). In the red area, scar states can occur. Below the red dashed line, *R*_c_ is smaller than the magnetic length *l*_*B*_ and Landau levels start to dominate the transport (light grey area). (**d**) Conductance as a function of antisymmetric gate tuning 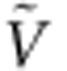 and magnetic field is shown in the lower panel, and its derivative d*G*/d*B* (in arbitrary units) in the upper panel. Striking lines of high and low conductance with a parabola-like B-dependence can be observed. (**e**) In a smooth p–n junction (here: linear *n*(*x*)), the cyclotron orbits become elongated along the *y* direction due to the additional electric field caused by the density gradient. (**f**) 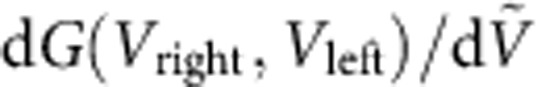 at 120 mT. The blue dashed lines in **d** and **f** are equivalent. The inset shows lines of constant electric field ***E***_*x*_ at the p–n interface as a function of *V*_right_,*V*_left_ taken from electrostatic simulations.

We now discuss the regime where snake states emerge. In Fig. 3a, a snake state at a sharp p–n junction is sketched. Consider a charge carrier trajectory starting at the grey cross with momentum **k** in −*x* direction. Due to the magnetic field, the trajectory is bent towards the p–n interface within the cyclotron diameter 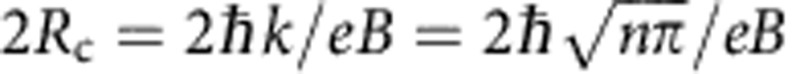. If the trajectory hits the p–n interface, the hole will be transmitted to the n side with high probability due to Klein tunnelling^20^. At the upper edge of the sample, the snake trajectory scatters at the left side, resulting in a current towards the left contact. At lower *n*, *R*_c_ is reduced. As sketched in Fig. 3b, the snake trajectory scatters to the right at the upper edge, resulting in a net current towards the right contact. With this mechanism, one expects conductance oscillations that depend on *B* and *n*, and constant conductance along curves where *R*_c_ is constant. In Fig. 3c, we display calculated functions *n*(*B*) for constant *R*_c_. Snake states occur once the cyclotron diameter 2*R*_c_ is smaller than the sample width *W* (green dashed curve) and can be described by quasiclassical trajectories as long as *R*_c_ is larger than the magnetic length 
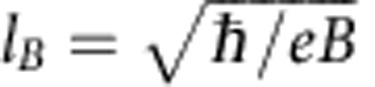
 (red dashed line). In the regime *W*>2*R*_c_>*l*_*B*_, additional parabolic lines show the condition for which the number of oscillations in the snake pattern is fixed and commensurate with *W*, that is, *m* × 2*R*_c_=*W* with *m*=1,2,3,....

In Fig. 3d, we show the measured conductance 
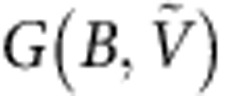
 (bottom) and its numerical derivative d*G*/d*B* (top). The measurement exhibits strong oscillations that follow parabola-like curves. We notice that the oscillations occur on a background of strongly decreasing conductance from *G*≈6*e*^2^/*h* to *G*<2*e*^2^/*h*. The steep decrease indicates that the transport becomes dominated by the low-density region close to the p–n interface and this happens when 2*R*_c_<*W*.

In a real p–n interface, the density does not sharply jump from the p to the n side but evolves smoothly. An electron trajectory in such a smooth p–n interface is sketched in Fig. 3e. Here the density gradient leads to an electric field *E*_*x*_ and its interplay with the perpendicular magnetic field results in **E** × **B** drift (here along *y*), leading to elongated cyclotron orbits. The condition *R*_*y*_=*const*. can be studied in the measurement of Fig. 3f, where we show 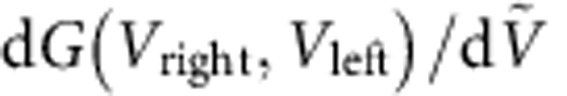 at *B*=120 mT. The measured oscillation pattern follows curves of constant *E*_*x*_ at the p–n interface (obtained from electrostatic simulations) as shown in the inset.

### Tight-binding theory of snake states

**Figure 4:**
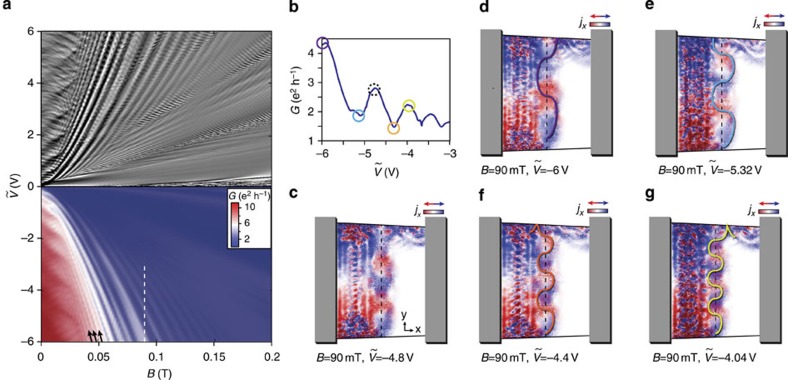
Tight-binding transport calculation reproducing the experimental results and local current density profiles revealing the snake states. (**a**) Tight-binding calculation of conductance 
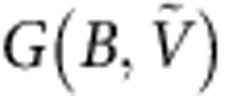
 (bottom) and its numerical derivative d*G*/d*B* (top, in arbitrary unit). The parabola-like lines seen in the experiment are well captured. (**b**) 
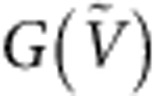
 along the white dashed line in **a** at *B*=90 mT. (**c**) Calculated *x* component of the local current density distribution, *j*_*x*_, for electrons injected from the left contact with a small d.c.-offset at *B*=90 mT and 
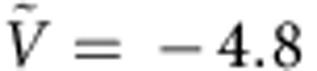
 V (dashed circle in **b**). The complex resonance pattern in the left cavity consists of ‘bubbling’ trajectories that do not contribute to conductance. At the p–n interface (dashed line), an alternating current pointing to the left (red) and right (blue) is observed. (**d**–**g**) Current density profiles at different 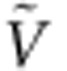 corresponding to the circles in **b**. As a guide to the eye, a snaking trajectory following *j*_*x*_ is added. From one conductance maximum to the next (that is, **c** to **g**), one snake period is added. The snake state in **e** and **f** corresponds to a conductance minimum and **c**,**d** and **g** to a maximum.

So far, we have seen that the oscillation pattern occurs in the regime where snake states are expected (that is, 2*R*_c_<*W*) and that the oscillations are related to transport along the p–n interface. We now present a quantitative comparison between experiment and theory. Figure 4a shows a quantum transport simulation of 
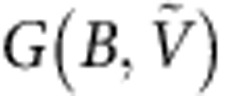
 and 
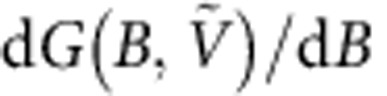
 on the basis of a scalable tight-binding model^19^ that fully takes into account the device geometry. The simulation compares very well with the measurement shown in Fig. 3d. The parabola-like patterns are reproduced and a similarly steep decrease of conductance is obtained. In Fig. 4b, a slice following the white dashed line in Fig. 4a is shown. The visibility Δ*G*/*G* of the oscillations reaches 30% in theory and experiment and is enabled by the strong Klein collimation at the smooth p–n interface.

Next we apply the Keldysh–Green’s function method to extract local current density profiles (see Methods) at high and low conductance along this line. In Fig. 4c, we show the *x* component of the current density *j*_*x*_, taken at 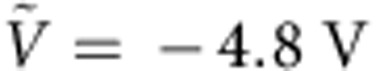 (dashed circle in Fig. 4b). The current is injected from the left contact using a small d.c. offset. In the left cavity, a complex resonance pattern appears, given by so-called ‘bubbling’ trajectories^21^, which are reflected before reaching the p–n interface and do not contribute to current between the contacts. The pattern relevant for transport is located at the p–n interface (dashed line). We observe that *j*_*x*_ changes sign along the p–n interface and that the blue and red regions penetrate the dashed line, indicating that transport is dominated by Klein-collimated snake trajectories. As a guide to the eye, we added a curve in Fig. 4d–g that follows the snake state. This is done for different 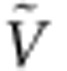 values for which *G* is maximal/minimal at a fixed magnetic field of 90 mT (coloured circles in Fig. 4b). In Fig. 4d, for example, the current density profile corresponds to a conductance maximum where the current points to the right at the upper edge of the p–n interface. One period is added by changing 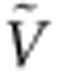 from one maximum to the next. More current density profiles evolving with 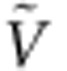 at fixed *B*=90 mT are shown in Supplementary Movie 1. By tracing along one of the parabola-like patterns, the current density profile of the snake state stays constant (an example is given in Supplementary Movie 2).

The conductance oscillates as a function of the ratio *W*/2*R*_*y*_, the exact snake period corresponding to 4*R*_*y*_ is, however, difficult to determine using quasiclassical trajectories, since current is injected from many points under various angles resulting in a complex cusp structure similar to what was predicted in refs 22, 23, 24, 25. The excellent agreement between measurement and calculated conductance for which we could determine local current density profiles clearly indicates that the oscillations result from snake state trajectories.

There are additional parabola-like structures at lower magnetic field indicated by arrows in Fig. 4a, these structures are, however, less pronounced in the experimental data. Those resonances occur in a regime where scar states (Fig. 1b) would be expected. The parabola-like behaviour indicates that the states are commensurate with a cavity dimension. In the model, the resonances disappear for non-reflective contacts as expected for scar states.

## Discussion

We investigated the magneto-conductance of a ballistic graphene p–n junction in different magnetic field regimes. We have observed resonance patterns occurring in the intermediate quasiclassical regime in experiment and theory, which result from the formation of snake states at the p–n interface. Among many other possibilities, these states can be used to guide electrons on arbitrary paths with a high efficiency even at very low magnetic fields. This could be used to guide electrons away from sample edges to suppress uncontrolled momentum or spin scattering. The directional scattering at the sample boundaries could be used to implement multi-terminal switches^24^,^26^. Furthermore, the similarity between Andreev reflection and Klein tunnelling is stressed in theory^27^ leading to a correspondence of snake states and Andreev edge states which are of theoretical^28^ and experimental^29^ interest. Our work points out that snake states are highly tunable and occur at low fields and that ballistic graphene p–n junctions in a magnetic field reveal novel and intriguing phenomena.

## Methods

### Experimental methods

High-quality graphene is obtained by *in situ* current annealing^30^. All the measurements were performed in a variable temperature Helium cryostat with a base temperature of 1.5 K. We measured differential conductance *G*=d*I*/d*V* by standard lock-in technique applying an a.c. voltage of 0.1 mV at 77 Hz.

For the quantum Hall data of Fig. 2b,c, we subtracted a contact resistance of 1.2 kΩ.

We extracted the cavity length *L* used in Fig. 2d,e from the spacing Δ*n* between resonant Fabry–Pérot peaks in the bipolar situation 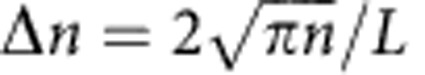 and obtained *L*≈0.8 μm.

### Simulation methods

Real-space Green’s function method in the tight-binding framework using a scaled graphene Hamiltonian^19^ is applied to simulate ballistic quantum transport in the present device, taking into account the realistic on-site energy profile obtained by three-dimensional electrostatic simulation for the self-partial capacitances of the bottom gates. All the presented conductance simulations are obtained by calculating the transmission function at zero temperature, combined with the contact resistance 1.2 kΩ. Local current densities are imaged by applying the Keldysh–Green’s function method in the linear response regime^31^ on the basis of the same model Hamiltonian used for conductance simulation. At each lattice site *n*, the bond charge current density **J**_*n*_=∑_*m*_**e**_*n*→*m*_‹*J*_*n*→*m*_› is computed, where the summation runs over all the sites *m* nearest to *n*, **e**_*n*→*m*_ is the unit vector pointing from *n* to *m*, and ‹*J*_*n*→*m*_› is the quantum statistical average of the bond charge current operator *J*_*n*→*m*_ (ref. 32). After computing for each site, the position-dependent current density profile **J**(*x*,*y*)=[*j*_*x*_(*x*,*y*),*j*_*y*_(*x*,*y*)] is imaged. In Fig. 4c–g, the *x* component *j*_*x*_(*x*,*y*) is shown.

The low-field Fabry–Pérot interference contours sketched in Fig. 2e are numerically obtained from solving the resonance condition Δ*Φ*=2*jπ*, arising from the path difference between the directly transmitted and twice reflected trajectories within the p cavity as sketched in the inset of Fig. 2e, which is found to be the major interference contribution. For such a simplified model, the phase difference is given by Δ*Φ*=*Φ*_WKB_+*Φ*_AB_+*Φ*_Berry_+*Φ*_0_, where 
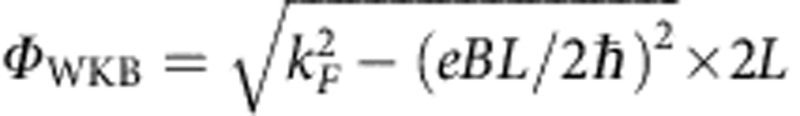
 is the kinetic WKB-phase, *Φ*_AB_=*δA* × *eB*/*ℏ* is the Aharanov–Bohm phase due to the flux enclosed by the bent orbit segments, 
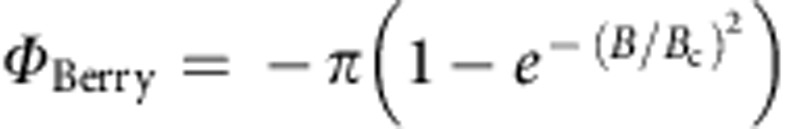
 is the Berry phase, and *Φ*_0_=*π* is a constant phase due to reflections off the two p–n junction interfaces of the p cavity (smooth at the middle and sharper at the contact side). Here *k*_*F*_ is the numerical average of 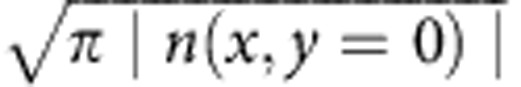 within the p cavity. The cavity size *L* is numerically determined and is about half of the flake length *L*/2=840 nm. The loop area is given by *δA*=*R*_c_^2^(*φ*−sin *φ*) with *φ*=2 arcsin (*L*/2*R*_c_) and *R*_c_=*ℏk*_*F*_/*eB*. The form of the Berry phase follows from the consideration of ref. 33 with the critical field estimated by 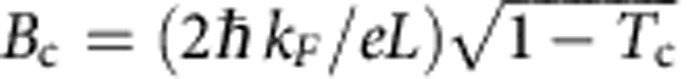, where the critical transmission value *T*_c_ is a parameter close to one and does not significantly influence the shape of the contours; *T*_c_=0.95 is chosen. The contours sketched in Fig. 2e correspond to *j*=1,2,⋯,8.

## Author contributions

P.R., P.M. and E.T. fabricated the devices. The measurements were performed by P.R., P.M., R.M., E.T. and M.W. M.-H.L. performed the simulations. C.S. and K.R. guided the work. All the authors worked on the manuscript.

## Additional information

**How to cite this article:** Rickhaus, P. *et al*. Snake trajectories in ultraclean graphene p–n junctions. *Nat. Commun*. 6:6470 doi: 10.1038/ncomms7470 (2015).

## Supplementary Material

Supplementary Movie 1 In this movie we show the local current density distribution in x- (jx) and y-direction (jy) for a fixed magnetic field of 90 mT. By tuning the densities in the p- and n- cavities (via \tilde V) the number of nodes of the snake state along the interface is changing, resulting in conductance oscillations.

Supplementary Movie 2 In this movie the density and magnetic field are changed such, that the conductance stays constant, i.e. we sweep along one of the parabola-like lines. Even though the pattern in the left cavity is strongly changing, the snake state along the interface remains unchanged.

## References

[b1] van Houten H. . Coherent electron focusing with quantum point contacts in a two-dimensional electron gas. Phys. Rev. B Condens. Matter 39, 8556–8575 (1989) . 9947569 10.1103/physrevb.39.8556

[b2] Taychatanapat T., Watanabe K., Taniguchi T. & Jarillo-Herrero P. Electrically tunable transverse magnetic focusing in graphene. Nat. Phys. 9, 225–229 (2013) .

[b3] Rickhaus P. . Ballistic interferences in suspended graphene. Nat. Commun. 4, 2342 (2013) . 23946010 10.1038/ncomms3342

[b4] Grushina A. L., Ki D.-K. & Morpurgo. A. F. A ballistic pn junction in suspended graphene with split bottom gates. Appl. Phys. Lett. 102, 223102 (2013) .

[b5] Huard B. . Transport measurements across a tunable potential barrier in graphene. Phys. Rev. Lett. 98, 236803 (2007) . 17677928 10.1103/PhysRevLett.98.236803

[b6] Özyilmaz B. . Electronic transport and quantum hall effect in bipolar graphene p-n-p junctions. Phys. Rev. Lett. 99, 166804 (2007) . 17995279 10.1103/PhysRevLett.99.166804

[b7] Gorbachev R. V., Mayorov A. S., Savchenko A. K., Horsell D. W. & Guinea F. Conductance of p-n-p graphene structures with "air-bridge" top gates. Nano Lett. 8, 1995–1999 (2008) . 18543979 10.1021/nl801059v

[b8] Ye P. D., Weiss D. & Gerhardts R. R. Electrons in a periodic magnetic field induced by a regular array of micromagnets. Phys. Rev. Lett. 74, 3013 (1995) . 10058081 10.1103/PhysRevLett.74.3013

[b9] Williams J. R. & Marcus C. M. Snake states along graphene p-n junctions. Phys. Rev. Lett. 107, 046602 (2011) . 21867027 10.1103/PhysRevLett.107.046602

[b10] Young A. F. & Kim P. Quantum interference and klein tunnelling in graphene heterojunctions. Nat. Phys. 5, 222–226 (2009) .

[b11] Bird J. . Lead-orientation-dependent wave function scarring in open quantum dots. Phys. Rev. Lett. 82, 4691–4694 (1999) .

[b12] Novoselov K. S. . Two-dimensional gas of massless dirac fermions in graphene. Nature 438, 197–200 (2005) . 16281030 10.1038/nature04233

[b13] Zhang Y., Tan Y.-W., Stormer H. L. & Kim P. Experimental observation of the quantum hall effect and berry's phase in graphene. Nature 438, 201 (2005) . 16281031 10.1038/nature04235

[b14] Williams J. R., Dicarlo L. & Marcus C. M. Quantum hall effect in a gate-controlled p-n junction of graphene. Science 317, 638–641 (2007) . 17600183 10.1126/science.1144657

[b15] Abanin D. A. & Levitov L. S. Quantized transport in graphene p-n junctions in a magnetic field. Science 317, 641–643 (2007) . 17600182 10.1126/science.1144672

[b16] Tombros N. . Large yield production of high mobility freely suspended graphene electronic devices on a polydimethylglutarimide based organic polymer. J. Appl. Phys. 109, 093702 (2011) .

[b17] Maurand R. . Fabrication of ballistic suspended graphene with local-gating. Carbon 79, 486–492 (2014) .

[b18] Cayssol J., Huard B. & Goldhaber-Gordon D. Contact resistance and shot noise in graphene transistors. Phys. Rev. B 79, 075428 (2009) .

[b19] Liu M.-H. . Scalable tight-binding model for graphene. Phys. Rev. Lett. 114, 036601 (2015) . 25659011 10.1103/PhysRevLett.114.036601

[b20] Cheianov V. & Falko V. Selective transmission of Dirac electrons and ballistic magneto-resistance of n-p junctions in graphene. Phys. Rev. B 74, 041403 (2006) .

[b21] Carmier P., Lewenkopf C. & Ullmo D. Semiclassical magnetotransport in graphene n-p junctions. Phys. Rev. B 84, 195428 (2011) .

[b22] Davies N. . Skipping and snake orbits of electrons: singularities and catastrophes. Phys. Rev. B 85, 155433 (2012) .

[b23] Patel A. A., Davies N., Cheianov V. & Fal'ko V. I. Classical and quantum magneto-oscillations of current flow near a p-n junction in graphene. Phys. Rev. B 86, 081413 (2012) .

[b24] Milovanovic S. P., Ramezani Masir M. & Peeters F. M. Spectroscopy of snake states using a graphene Hall bar. Appl. Phys. Lett. 103, 233502 (2013) .

[b25] Milovanovic S. P., Ramezani Masir M. & Peeters F. M. Magnetic electron focusing and tuning of the electron current with a pn-junction. J. Appl. Phys. 115, 043719 (2014) .

[b26] Chen J.-C., Xie X. C. & Sun Q.-f. Current oscillation of snake states in graphene p-n junction. Phys. Rev. B 86, 035429 (2012) .

[b27] Beenakker C., Akhmerov A., Recher P. & Tworzydlo J. Correspondence between Andreev reflection and Klein tunneling in bipolar graphene. Phys. Rev. B 77, 075409 (2008) .

[b28] Hoppe H., Zulicke U. & Schon G. Andreev reflection in strong magnetic fields. Phys. Rev. Lett. 84, 1804–1807 (2000) . 11017630 10.1103/PhysRevLett.84.1804

[b29] Rickhaus P., Weiss M., Marot L. & Schonenberger C. Quantum hall effect in graphene with superconducting electrodes. Nano Lett. 12, 1942–1945 (2012) . 22417183 10.1021/nl204415s

[b30] Moser J., Barreiro A. & Bachtold. A. Current-induced cleaning of graphene. Appl. Phys. Lett. 91, 163513 (2007) .

[b31] Cresti A., Grosso G. & Parravicini G. Electronic states and magnetotransport in unipolar and bipolar graphene ribbons. Phys. Rev. B 77, 115408 (2008) .

[b32] Nikolic B., Zarbo L. & Souma S. Imaging mesoscopic spin Hall flow: spatial distribution of local spin currents and spin densities in and out of multiterminal spin-orbit coupled semiconductor nanostructures. Phys. Rev. B 73, 075303 (2006) .

[b33] Shytov A., Rudner M. & Levitov. L. Klein backscattering and Fabry-Perot interference in graphene heterojunctions. Phys. Rev. Lett. 101, 156804 (2008) . 18999625 10.1103/PhysRevLett.101.156804

